# HERC1 E3 Ubiquitin Ligase Is Necessary for Autophagy Processes and for the Maintenance and Homeostasis of Vesicles in Motor Nerve Terminals, but Not for Proteasomal Activity

**DOI:** 10.3390/ijms26020793

**Published:** 2025-01-18

**Authors:** Miguel Ángel Pérez-Castro, Francisco Hernández-Rasco, Isabel María Alonso-Bellido, María S. Letrán-Sánchez, Eva María Pérez-Villegas, Joana Vitallé, Luis Miguel Real, Ezequiel Ruiz-Mateos, José Luis Venero, Lucía Tabares, Ángel Manuel Carrión, José Ángel Armengol, Sara Bachiller, Rocío Ruiz

**Affiliations:** 1 Institute of Biomedicine of Seville (IBiS), Virgen del Rocío University Hospital/CSIC/University of Seville, 41013 Seville, Spain; 2 Department of Biochemistry and Molecular Biology, School of Pharmacy, University of Seville, 41012 Seville, Spain; 3 Center for Molecular and Systems Biology, Lunenfeld-Tanenbaum Research Institute, Mount Sinai Hospital, Toronto, ON M5G 1X5, Canada; 4 Department of Physiology, Anatomy and Cellular Biology, University of Pablo de Olavide, 41013 Seville, Spain; 5 Clinical Unit of Infectious Diseases, Microbiology and Parasitology, Laboratory of Immunovirology, Virgen del Rocío University Hospital, 41013 Seville, Spain; 6 Unit of Infectious Diseases and Microbiology, Institute of Biomedicine of Seville (IBiS), University Hospital of Valme/CSIC/University of Seville, 41013 Seville, Spain; 7 Centro de Investigación Biomédica en Red de Enfermedades Infecciosas (CIBERINFEC), 28029 Madrid, Spain; 8 Department of Medical Biochemistry, Molecular Biology and Immunology, School of Medicine, University of Seville, 41009 Seville, Spain; 9 Department of Medical Physiology and Biophysics, University of Seville, 41009 Seville, Spain

**Keywords:** proteasome, neuromuscular junction, autophagy, vesicles, synapses

## Abstract

The ubiquitin proteasome system (UPS) is implicated in protein homeostasis. One of the proteins involved in this system is HERC1 E3 ubiquitin ligase, which was associated with several processes including the normal development and neurotransmission at the neuromuscular junction (NMJ), autophagy in projection neurons, myelination of the peripheral nervous system, among others. The tambaleante (tbl) mouse model carries the spontaneous mutation Gly483Glu substitution in the HERC1 E3 protein. Using this model, we analyzed the implication of HERC1 E3 ubiquitin ligase in the activity of UPS, autophagy, and synaptic homeostasis in brain and muscle tissues. Regarding UPS, no differences were found in its activity nor in the specific gene expression in both brain and muscle tissues from tbl compared with the control littermates. Furthermore, the use of the specific UPS inhibitor (MG-132), did not alter the evoked neurotransmitter release in the levator auris longus (LAL) muscle. Interestingly, the expression of the autophagy-related gene p62 was significantly increased in the muscle of tbl compared to the control littermates. Indeed, impaired evoked neurotransmitter release was observed with the autophagy inhibitor Wortmannin. Finally, altered levels of Clathrin and Synaptophysin were detected in muscle tissues. Altogether, our findings show that HERC1 E3 ubiquitin ligase mutation found in tbl mice alters autophagy and vesicular recycling without affecting proteasomal function.

## 1. Introduction

Mutations in HERC1 (HECT domain and RCC1 domain) E3 ubiquitin ligase were related to different human pathologies such as neuromuscular disorders, Parkinson’s disease, autism spectrum disorder, X-linked retinitis pigmentosa, and juvenile amyotrophic lateral sclerosis [1]. However, the Online Mendelian Inheritance in Man (OMIM) database [2] lists only one genetic disease that appears linked to inherited mutations in the HERC1 gene: macrocephaly, dysmorphic facies, and psychomotor retardation (MDFPMR, #617011) [3,4,5,6]. The tbl mice model was described for the first time to be associated with an ataxia phenotype in 1987 due to adult cerebellar death of Purkinje neurons [7,8]. Tbl mice harbor a single nucleotide transition within the Herc1 gene at position 1448, giving rise to a mutated protein with a Gly483Glu amino acid substitution that is less prone to degradation [9]. The specific mutation in tbl mice is located in the regulator of the chromosome condensation 1 (RCC1)-like domain (RDL) of HERC1 [9]. It is important to note that none of the mutations described in the human disease MDFPMR are located in this region [3,4,5,6]. Most of the mutations are associated with the HECT region or related to the generation of truncated forms of the HERC1 protein [1]. In the case of Schwarz et al., the mutation is associated with the HECT region, resulting in a gain-of-function state of the protein [5].

Studies have focused on finding out which is/are the substrates of this E3 ubiquitin ligase and/or in which cellular processes were involved. The importance of these investigations was to be expected given its involvement in Purkinje cell survival [7,10]. HERC1 contains multiple domains involved in different processes. For example, while the HECT domain is the catalytic one involved in the ubiquitination of target proteins [11], HERC1 RLD domains interact with several proteins (CTL, ARF, and Rab) and with phosphoinositides, which are involved in membrane trafficking (for review, see [1,12]). Additionally, HERC1 interacts with the tuberous sclerosis complex (TSC) 2 protein, which may act as a regulator of mTOR (mammalian target of rapamycin) complex 1 (mTORC1) kinase activity [13]. Recently, HERC1 was identified as a quality-control factor that monitors failures in proteasome assembly through the degradation of unassembled PSMC5 (26S proteasome AAA-ATPase subunit Rpt6) [14].

Apart from cerebellar-associated ataxia, additional series of phenotypes were described by our group. It was first described that the alteration of motor performance, associated with an impaired evoked neurotransmitter release at the neuromuscular junction (NMJ) in the levator auris longus (LAL), tranversus abdominis (TVA), and gastronemius (GN) muscles, occurred one month before the onset of the tbl phenotype [15]. Afterwards, we reported increased autophagy in projection neurons without cell loss as severe as Purkinje cells [16]. Interestingly, a relationship was identified between Herc1 overexpression and the anomalous myelination of the sciatic nerve thick and thin axons along with alterations in non-myelinating terminal Schwann cells at NMJ [17]. Additionally, tbl mice exhibited impaired associative learning due to the absence of long-term potentiation (LTP), which was associated with dendritic spine alterations [18,19]. Finally, in vitro cultured hippocampal neurons exhibited altered membrane dynamics of the presynaptic terminal, disrupting the homeostasis of synaptic vesicle recycling [20]. Along this line, Montes-Fernández et al. have analyzed these two processes in vitro [20], focused on the described role of Herc1 through its C-terminal RCC1 domain (RLD2), which forms a ternary complex with Clathrin (CLT) and the heat shock protein 70 [21]. In summary, the primary alterations associated with Herc1 mutation are related to autophagy [9,16] and defective synaptic function [15,17]. To date, no connection has been identified between the tbl phenotypes and their role as ubiquitin ligases, nor with changes in proteasomal activity. Therefore, whether these alterations arise from dysfunction in proteasome activity or a ubiquitin ligase-independent Herc1 function remains under debate [1,20]. To further explore this matter, in this study, we evaluated proteasome activity, and the expression of key genes involved in the ubiquitin proteasome system (UPS), autophagy, and synaptic processes of control and tbl mice by using real-time qPCR. Finally, we analyzed whether the alterations observed in the vesicular recycling process in muscle [15] and neurons [20] may occur in a similar way in the LAL muscle in the tbl mouse model by employing specific inhibitors of autophagy and proteasome activity. Our findings strongly support that the tbl mutation compromises autophagy and vesicular recycling while sparing proteasomal function.

## 2. Results

### 2.1. Accumulation of Herc1 Protein Does Not Affect UPS Activity in tbl Mutant Mice

**Figure 1:**
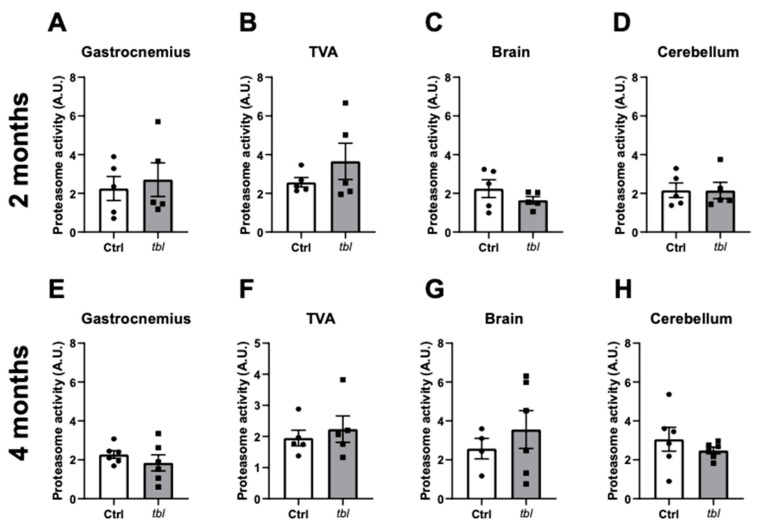
Proteasome activity in Ctrl and tbl mutant mice. Chymotrypsin proteasome activity in (**A**,**E**) gastrocnemius and (**B**,**F**) TVA muscles, and (**C**,**G**) brain and (**D**,**H**) cerebellum of control (Ctrl) and tambaleante (tbl) mutant mice at two (upper graphs) and four (lower graphs) months of age. n = 5–6 animals/group; technical duplicates. White bars represent Ctrl mice; gray bars represent tbl mice, and individual dots represent individual mice. Data are represented as mean ± SEM; *p* > 0.05.

Gly483Glu substitution within Herc1 induces protein accumulation, leading to the tbl phenotype in mice [9]. Chymotrypsin proteasomal activity analyses were performed here in GN, TVA, brain, and cerebellum to analyze the role of Herc1 in the ubiquitin proteasome system. No significant differences were observed in any of the tissues analyzed at either two months (Figure 1A–D) or four months of age (Figure 1E–H).

### 2.2. The Proteasome Inhibitor MG-132 Does Not Affect Neurotransmission in the LAL Muscle

**Figure 2:**
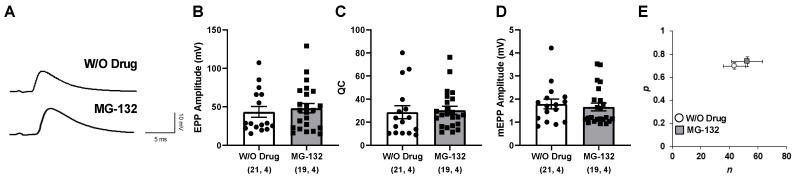
No changes in evoked neurotransmitter release following MG-132 application in Ctrl LAL muscle. (**A**) Representative EPP traces, (**B**) mean EPP amplitudes, (**C**) mean quantum content (QC), and (**D**) mean of mEPP in vehicle (W/O drug) and MG-132-treated LAL muscles. (**E**) *n* (number of occupied sites) and *p* (probability of release) estimated in W/O drug and MG-132-treated LAL muscles. [(n, N) n, number of fibers; N, number of mice]; *p* > 0.05.

One of the main features observed in intracellular recordings of the LAL muscle in the tbl mice was the decrease in neurotransmitter release [15], reflected by the reduction in both the size of the EPP and the QC, potentially associated with a reduction in the number of release sites, as calculated by binomial analysis. To investigate whether the impairment in neurotransmission depends on proteasome activity deficiency in tbl, intracellular recordings were performed in the LAL muscle of the control mice using the specific proteasome inhibitor MG-132. No significant differences were detected in the amplitude of EPP, mEPP, or QC (Figure 2A–D) nor in the estimated number of release sites or in the probability of release (Figure 2E). These results suggest that proteasome activity does not play a predominant role in neurotransmitter release.

### 2.3. UPS Gene Expression Is Not Altered in TVA Muscle of tbl Mutant Mice

**Figure 3:**
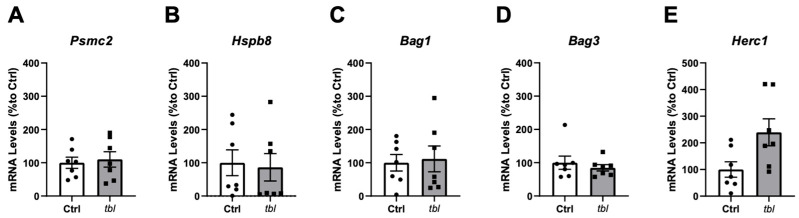
RT-qPCR expression of genes related to UPS in TVA muscle from Ctrl and tbl mutant mice. mRNA levels (% to Ctrl) of (**A**) Psmc2, (**B**) Hspb8, (**C**) Bag1, (**D**) Bag3, and (**E**) Herc1. n = 7 animals/group; average of technical and experimental triplicates. White bars represent Ctrl mice; gray bars represent tbl mice; individual dots represent individual mouse values. Data are represented as mean ± SEM; *p* > 0.05.

Although we did not find differences in the proteasome activity between tbl mice and their littermate controls in different tissues (Figure 1), we wondered if genes involved in UPS function could be altered. No significant differences were found in the expression levels of Psmc2 (Figure 3A), Hspb8 (Figure 3B), Bag1 (Figure 3C), Bag3 (Figure 3D), and Herc1 (Figure 3E).

### 2.4. Autophagy-Related Gene p62/SQSTM1 Is Upregulated in tbl TVA Muscle

**Figure 4:**
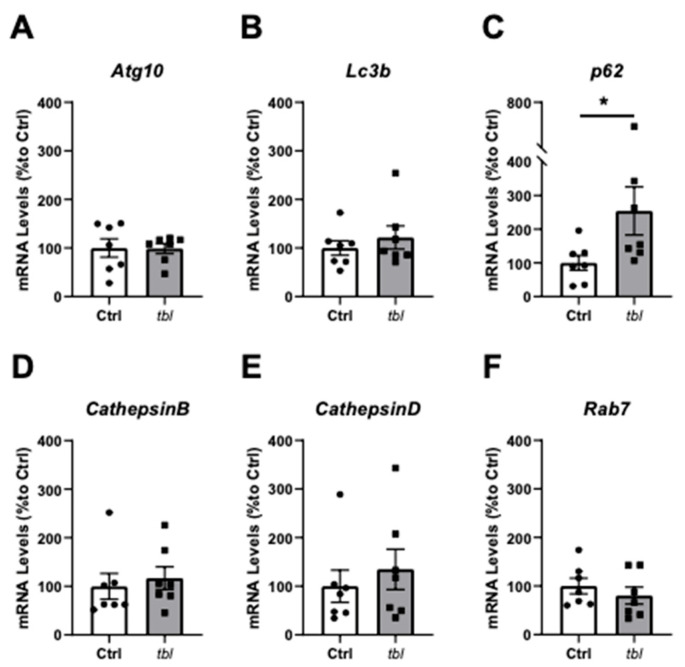
RT-qPCR expression analysis of genes related to autophagy pathway in TVA muscle from Ctrl and tbl mutant mice. mRNA levels (% to Ctrl) of (**A**) Atg10, (**B**) Lc3b, (**C**) p62, (**D**) CathepsinB, (**E**) CathepsinD, and (**F**) Rab7. n = 7 animals/group; technical triplicates. White bars represent Ctrl mice; gray bars represent tbl mice; individual dots represent one mouse. Data are represented as mean ± SEM. * *p* < 0.05.

Autophagy alterations were previously reported in tbl mutant mouse brain [16]. To further characterize this process and its function in muscle tissue, we analyzed the gene expression of key markers involved in autophagosome formation and lysosomal fusion (for review, see [22]). No significant changes were detected in the expression of Atg10 (Figure 4A), Lc3b (Figure 4B), CathepsinB (Figure 4D), CathepsinD (Figure 4E), or Rab7 (Figure 4F). However, the expression of p62 was significantly elevated in tbl mice compared to their Ctrl littermates (Figure 4C).

### 2.5. Neurotransmitter Release Decreases Following Treatment with Autophagy Inhibitor Wortmannin

**Figure 5:**
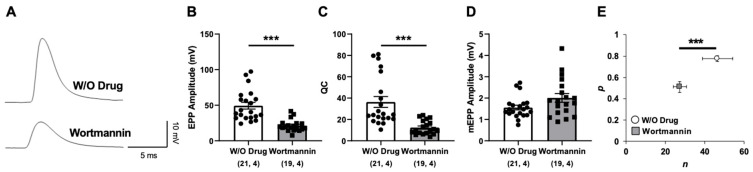
Impaired evoked neurotransmitter release after Wortmannin application in Ctrl LAL muscle. (**A**) Representative EPP traces, (**B**) mean EPP amplitudes, (**C**) mean quantum content (QC), and (**D**) mean of mEPPs in vehicle (W/O Drug) and Wortmannin-treated LAL muscles. (**E**) *n* (number of occupied sites) and *p* (probability of release) estimated in vehicle (W/O Drug) and Wortmannin-treated LAL muscles. [(n, N) n, number of fibers; N, number of mice]. Data are represented as mean ± SEM. *** *p* < 0.001.

Given the altered expression of autophagy-related genes in tbl mutant mice but the absence of changes in proteasome activity (Figure 1, Figure 2 and Figure 3), we investigated whether autophagy inhibition would alter the neurotransmission in the control LAL muscle. Following the same protocol as performed in Figure 2, we observed that after incubation with the autophagy inhibitor Wortmannin for 30 min, the EPP amplitude and QC were significantly decreased (Figure 5B and Figure 5C, respectively). No difference was found in mEPP amplitudes, indicating that the quantal size remained unaffected by this treatment (Figure 5D). Similarly, a significant decrease in the number of available release sites was detected (Figure 5E). Noteworthy, the alteration in the neurotransmitter release elicited after the application of Wortmannin closely resembled that observed in the tbl model [15].

### 2.6. Altered Levels of Clathrin and Synaptophysin Are Found at NMJ of tbl Mutant Mice

**Figure 6:**
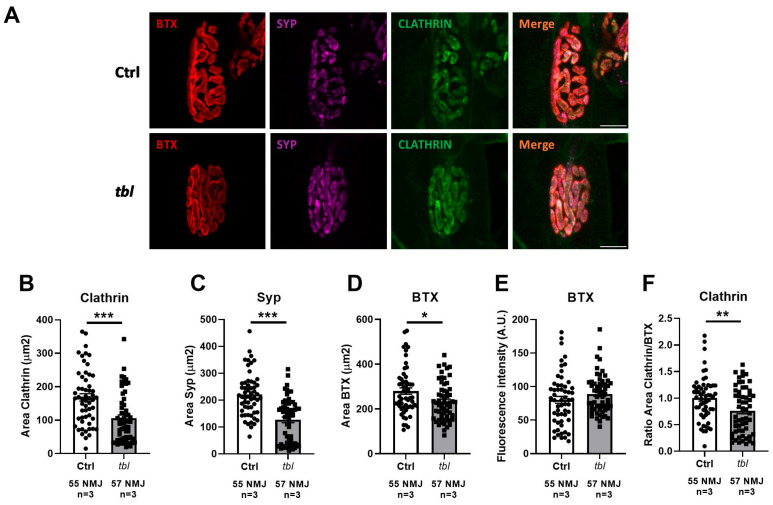
BTX, Syp, and Clathrin immunolabeling at NMJ of tbl and Ctrl mice at 2 months old. (**A**) Representative z-stack projections of confocal images of NMJs from LAL muscles stained with BTX-Rho (red), anti-Syp (magenta), and anti-Clathrin (green). Area measurement (µm^2^) of (**B**) Clathrin, (**C**) Syp, and (**D**) BTX. (**E**) Fluorescence intensity (A.U.) of BTX. (**F**) Ratio of Clathrin/BTX area. Scale bar: 10 μm. 55–57 NMJ were analyzed; n = 3 animals/group. Each dot represents one NMJ. White bars represent Ctrl mice; gray bars represent tbl mice. Data are represented as mean ± SEM. * *p* < 0.05; ** *p* < 0.01; *** *p* < 0.001.

Previous in vitro experiments demonstrated a dysregulation of presynaptic membrane dynamics in hippocampal neurons of tbl mice [20]. To further explore this phenomenon at the NMJ, we performed immunostaining for Clathrin (CTL) and Synaptophysin (Syp) in the LAL muscle of the Ctrl and tbl mice (Figure 6A). A significant reduction in the CTL area was observed at the NMJ of the tbl mice (Figure 6B). A similar result was also found for the Syp area (Figure 6C), as is consistent with our prior findings [15]. Although we confirmed that the BTX-labeled postsynaptic area was significantly smaller in the tbl mice (Figure 6D) [15], no absolute differences in the BTX fluorescence intensity were detected (Figure 6E). This indicates that the permeability of the muscles to the antibodies was similar between both groups. On the other hand, the ratio of the Clathrin/BTX area was significantly lower in the tbl mice, which may indicate impaired innervation of NMJ (Figure 6F).

### 2.7. Defects in Synaptic Gene Expression Are Found in tbl Mutant Mouse Muscle

**Figure 7:**
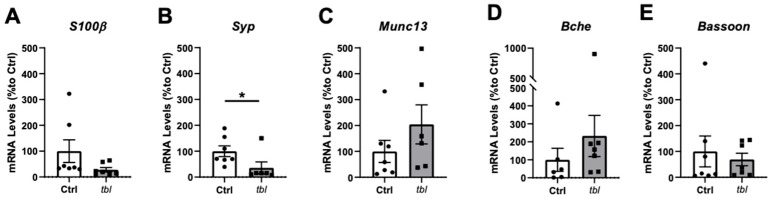
RT-qPCR gene expression analysis of synaptic components in TVA muscle. mRNA levels (% to Ctrl) of (**A**) S100β, (**B**) Syp, (**C**) Mun13, (**D**) Bche, and (**E**) Bassoon. n = 6–7 animals/group; technical triplicates. White bars represent Ctrl mice; gray bars represent tbl mice; individual dots represent one mouse. Data are represented as mean ± SEM. * *p* < 0.05.

Recently, we have demonstrated the importance of HERC1 in the regulation of presynaptic membrane dynamics in in vitro hippocampal neurons [20] as well as for normal muscle function and neurotransmitter release [15]. Therefore, we analyzed the expression of genes involved in synaptic function in the TVA muscle. No significant differences were found in the expression of S100β (Figure 7A), Munc13 (Figure 7C), Bche (Figure 7D), or Bassoon (Figure 7E). However, a significant reduction in mRNA levels of Syp (Figure 7B) was determined in the tbl TVA muscle in comparison with their Ctrl littermates (*p* = 0.02). These findings align with the previously observed reduction in the Syp area through immunofluorescence (Figure 6C).

## 3. Discussion

We present evidence that the overexpression of the mutated form of Herc1 (Gly483Glu) does not affect UPS activity in tbl mutant mice. Notably, we have not detected any change in neurotransmission using the proteasome inhibitor MG-132, confirming that the NMJ impairment identified in tbl mutant mice does not result from proteasome inhibition. Second, we confirm that the autophagy pathway is altered in the tbl TVA muscle through p62/SQSTM1. Furthermore, we observe a strong effect in neurotransmission release in the control LAL muscle using autophagy inhibitor Wortmannin, similar to what was observed in the tbl mice. Finally, we confirm that the areas of CTL and Syp are altered at the NMJ of the tbl mutant mice along with decreased expression of Syp. All these data lead us to support our hypothesis that changes observed at the NMJ of tbl mice are due to alterations in the autophagy and vesicular recycling system and not to a proteasome dysfunction.

The involvement of HERC1 in several cellular processes and its role in the survival of Purkinje neurons, together with the description of mutations associated with human diseases, makes it essential to decipher which pathway is involved and which is not in the tbl mouse phenotype. As HERC1 belongs to the E3 ubiquitin ligase family [23], first we evaluated its role in proteasome activity in different tissues in the tbl model. No differences in the proteasome activity were found in any of the tissues studied (Figure 1). The lack of differences in proteasomal activity in the muscles does not necessarily indicate that it is unaltered or that it does not impact neurotransmission, as the homogenate dissipates the specific molecular components of the neuromuscular junction. Therefore, future experiments should be conducted to ascertain whether there is a specific proteasomal impairment at the neuromuscular junction. Interestingly, the UPS14 (deubiquitinating enzyme USP14) mice model exhibits a similar phenotype to the tbl mice both in terms of Purkinje cell death and the impairment of neuromuscular function [24]. Remarkably, UPS 14 might have a different function to that of proteasome catalytic activity [25,26], similar to what we reported in tbl mutant mice. However, Herc1 was recently identified as a key player of a quality-control protein that monitors failures during proteasome assembly, specifically by mean of ubiquitination of PSMC5-PAAF1 complex in the aneuploidy breast cancer cell line MCF7 [14]. In fact, it seems that the presence of the tbl mutation leads to a failure in the control of the 19S subunit of the proteasome, provoking the accumulation of this aberrant protein causing the tbl phenotype. Although this fact cannot be completely ruled out, it is not unreasonable to think that the accumulation of a subunit of the proteasome is not enough to induce changes in the proteasome activity as we found in this work in different tissues (Figure 1). Moreover, we did not find alterations in the expression of different genes involved in UPS in tbl compared to the ctrl littermates (Figure 2). Importantly, altering the phosphorylation of this proteasome subunit in mouse models, which changed the catalytic rate of substrate degradation by the 26S proteasome, literally had no impact on synaptic plasticity [27]. Nevertheless, future experiments are needed to clarify this aspect.

On the other hand, neurotransmission at the NMJ in tbl mice is characterized by a decrease in the number of vesicles available [15], suggesting that Herc1 has an essential role in vesicle maintenance dynamics. To investigate the role of the proteasome in this dysfunction, intracellular recordings in control muscles using proteasome inhibitor MG-132 were performed (Figure 2). No differences were found in any of the neurotransmitter parameters analyzed after MG-132 administration in the control LAL muscle. However, in Drosophila NMJ using proteasome inhibitors [28] and in cultures of hippocampal neurons [29], an increase in neurotransmitter release with no change in the probability of release was described. This difference could be due to the model system in which the recording was made; however, future experiments are needed to clarify this aspect. Taken together, these findings could indicate that altered proteasome activity is unlikely the cause of the tbl phenotype.

Since our experimental data argues against a significant role of proteasomal activity in the tbl phenotype, we focus our research on another of the processes in which Herc1 has been implicated: autophagy. In fact, lower mTOR catalytic activity was already described in the cerebellum of tbl mice [9,17]. mTOR controls the autophagy flow, so it could be expected that this process would be altered. Increased levels of autophagosome markers were found in the tbl cerebellum [9] as well as in tbl projection neurons [16]. In the present work, markers of autophagy in the TVA muscle were measured by qPCR, showing altered autophagy throughout the increased expression of p62/SQSTM1 in tbl mice (Figure 4C). The overexpression of p62 may result from a compensatory effect. It is well-established that the mutation present in the tbl mice leads to increased levels of autophagy [1,9,16]. Furthermore, it was noted that the association of p62 with mTORC1 inhibits autophagy [30]. Therefore, the increase in gene expression could be a compensatory mechanism aimed at reducing autophagy levels in the tbl model. Interestingly, mutations in p62/SQSTM1 in patients with amyotrophic lateral sclerosis were described [31,32]. In addition, the overexpression of this protein was recently related to neuronal death [33]. To confirm the importance of autophagy processes in the release of neurotransmitter at the NMJ, we used an intracellular recording of the control muscles using the autophagy inhibitor Wortmannin. The observed effect mirrored those in the tbl model [34], supporting the hypothesis that defects in NMJ neurotransmission in tbl mice arise from autophagy alterations. In fact, the key role of autophagy in presynaptic function is widely known (for review see [35]). It would be highly interesting to use other specific autophagy inhibitors targeting downstream levels, such as ULK inhibitors. Future experiments involving these inhibitors would allow us to pinpoint the exact stage of the autophagic pathway that specifically affects neurotransmitter release at the NMJ. Therefore, it is plausible that some synaptic proteins could be dysregulated in tbl mice, in comparison with the control littermates, as we found in our analysis of the NMJ (Figure 6). Recently, Montes-Fernandez et al. described an alteration of the normal dynamic of excitatory presynaptic terminals in hippocampal neurons from tbl mice associated with changes in HERC1-CLT interaction and, as a consequence, interfering with the normal synaptic function [20].

Our study has some limitations. While the sample size was relatively small, we performed several experiments that could potentially complement our findings. Most of our experiments were focused on 4-month old mice, an age previously identified as representative of the characteristic phenotype of tbl mice [15,17]; therefore, future studies exploring more advanced ages would fully explain the underlying mechanisms of HERC1 E3-ubiquitin ligase in proteasome activity. Also, we cannot exclude the possibility of the effects of some compensatory mechanisms on proteasome activity during the progression of the tbl phenotype. Due to limited material, not all experiments were performed on every muscle type; however, the use of different muscles may provide a general overview of the various processes. Additionally, we only included male mice for a more homogenous group, as we did not find significant sex-based differences in our previously published papers; further research including females would provide valuable insights.

In conclusion, our findings indicate a primary role of Herc1 in autophagy processes and its essential role in the maintenance and homeostasis of synaptic vesicles in the NMJ, without any effect on proteasomal activity. Considering that most of the disorders found in humans with HERC1 mutations mimicked the tbl phenotype, these results may lead us to find new therapeutic targets and focus on the study of the pathways specifically involved.

## 4. Materials and Methods

### 4.1. Animals

Tambaleante (tbl) mutant mice were obtained by breeding pairs of carrier mice and genotyped by PCR, as previously described [9]. The mice were weaned at postnatal day 30 and group-housed (4–5 animals/cage) in standard cages on a 12 h light/dark cycle. Food, water, and nesting material were provided ad libitum. Experiments were conducted on males and age-matched littermate control (Ctrl) and mutant mice (tbl) at 2 and 4 months old. All experimental animal protocols in the present study were in accordance with the Guidelines of the European Union Council, following the Scientific Committee of Instituto de Investigación y Formación Agraria y Pesquera, Consejería de Agricultura, Pesca, Agua y Desarrollo Rural of Junta de Andalucía (Spain; 20/12/2017/174).

### 4.2. Immunofluorescence

Immunofluorescence of levator auris longus (LAL) muscles was performed as previously described [15]. First, the mice were sacrificed by exsanguination after being anesthetized with tribromethanol 2% (Sigma-Aldrich, Burlington, MA, USA). The LAL muscle was dissected out and incubated for 30 min in 4% paraformaldehyde (PFA, Panreac, Barcelona, Spain) and then in a solution of 0.1 M glycine in PBS for another 30 min. Then, the tissues were permeabilized with 1% (*v*/*v*) Triton X-100 (PBS-T 1%) for 1 h and incubated with the blocking solution (PBS, 5% BSA, PBS-T 1%) for 1 h. The muscles were then incubated overnight at 4 °C with primary antibodies (Synaptophysin, SYP, 1:500, Santa Cruz Biotechnology, Dallas, TX, USA; Clathrin, CTL, 1:500, Synaptic Systems, Göttingen, Germany). The following day, the muscles were rinsed for 1 h in PBS-T 0.05%, incubated with the corresponding secondary antibodies (donkey–anti-rabbit Alexa Fluor 647, donkey anti-mouse Alexa Fluor 488, 1:500, ThermoFisher, Waltham, MA, USA) and 10 ng/mL rhodamine-BTX (Sigma-Aldrich) for 1 h and rinsed again with PBS-T 0.05% for 90 min. Finally, the muscles were mounted with glycerol 50% in PBS for visualization. Images were taken using a Leica DM2500 confocal laser scanning microscope (Wetzlar, Germany) with a 63X oil-immersion objective and with a numerical aperture of 1.3. All acquisition parameters were kept constant for the Ctrl and tbl mutant mice and were taken by the same researcher, blinded to the genotype, and usually on the same day. The fluorescently labeled structures were offline analyzed using Fiji Image J software v1.53t (W. Rasband, National Institutes of Health, Bethesda, MD, USA).

### 4.3. Protein Extraction

Gastrocnemius (GN) and tranversus abdominis (TVA) muscles, brain, and cerebellum were dissected and homogenized by sonication (15 s) in 1 mL of cold lysis buffer (10 mM Tris-HCl, pH 7.8; 0.5 mM DTT; 5 mM MgCl_2_). Then, the samples were centrifuged at 400× *g*, 4 °C for 10 min. A protein supernatant was mixed with 20% of glycerol and stored at −80 °C until further use. Protein quantification was performed using the BioRad protein assay (Bio-Rad Laboratories, Inc., Hercules, CA, USA).

### 4.4. Proteasome Activity

Chymotrypsin-like proteasome activity was performed as described previously [36]. Briefly, protein extracts were incubated at 37 °C in 50 µL of lysis buffer containing 12.5 µg of a protein extract, 5 mM ATP (AppliChem, GmBH, Darmstadt, Germany), 50 mM EDTA (Sigma, Burlington, MA, USA), and 5 µM of the fluorogenic substrate Succ-LLVY-AFC (N-Succinyl-Leu-Leu-Val-Tyr 7-Amido 4 trifluormethylcoumarin, Sigma Aldrich). The samples were measured using Varioskan Flash (Thermo Fisher Scientific, Waltham, MA, USA) at 460 nm (one measurement every 5 min during 1 h).

### 4.5. Electrophysiology

Ex vivo electrophysiological recordings with microelectrodes of the LAL muscle fibers were performed as previously described [15]. First, the LAL muscle was isolated and pinned to a 2 mL cured silicone rubber chamber (Sylgard, Dow Corning, Midland, MI, USA). The LAL muscle was continuously perfused with a solution containing 125 NaCl, 5 KCl, 2 CaCl_2_, 1 MgCl_2_, 25 NaHCO_3_, and 30 glucose (all units, mM). Continuous flow of 95% O_2_ and 5% CO_2_ was applied to the solution to keep its pH at 7.35. All the recordings were performed at room temperature (22–23 °C).

### 4.6. Ex Vivo Electrical Stimulation and Intracellular Recordings

Electrical stimulation was performed as described previously [15,37]. Briefly, 0.2 ms square-wave pulses (2–40 V of amplitude and 0.5 Hz of frequency) were applied to the nerve and evoke end-plate potentials (EPPs) and miniature EPPs (mEPPs) were recorded. Pharmacological drugs were used to block, specifically the following: (i) skeletal muscle voltage-gated sodium channels, using µ-conotoxin GIIIB (2–4 µM, Alomone Labs, Jerusalem, Israel); (ii) 26S UPS, using MG132 (50 µM; Sigma Aldrich) [29]; and (iii) autophagy, using Wortmannin (1 µM; Alomone Labs, Jerusalem, Israel) [38]. Intracellular recordings were analyzed as described previously [15]. First, in each NMJ, the mean amplitude of EPP and mEPP were linearly normalized to a −70 mV resting membrane potential and EPP amplitudes were corrected for non-linear summation as conducted previously [39]. Then, the mean quantal content (QC) of each fiber was estimated by dividing the mean EPP amplitude by the mean mEPP amplitude [15,37].

### 4.7. RNA Extraction and RT-qPCR Analysis

At 4 months of age, the Ctrl and tbl mice were anesthetized with tribromethanol 2% and sacrificed by exsanguination. Total RNA from the TVA muscle was extracted using TRIsure (Bioline, Almería, Spain) following the manufacturer’s instructions. RNA concentrations were measured using Nanodrop (ND-1000, Thermo Fisher Scientific, Waltham, MA, USA) and 500 ng of total RNA was converted to a cDNA using Revertaid cDNA synthesis kit (Thermo Fisher Scientific, Waltham, MA, USA). Real-time qPCR (RT-qPCR) was performed using the listed primer sequences (Table 1). Amplification was conducted using the real-time PCR LightCycler 480 (Roche, Basel, Switzerland). Relative gene expression was normalized to mRNA levels of the housekeeping gene Gapdh. Data are represented as a percentage related to the levels of the Ctrl mice.

### 4.8. Statistics

Statistical analyses were performed using GraphPad Prism 8.0 Software for Macintosh (GraphPad Software, San Diego, CA, USA). Student’s *t*-test was used to compare quantitative variables between the tbl and Ctrl groups. In the graphs, the individual dots represent a single mouse. The data are reported as mean ± SEM. *p* values < 0.05 were considered statistically significant and are reported in the figure legends.

## Figures and Tables

**Table 1:** List of primers used in study. Gene identification (ID) numbers were reviewed at https://ncbi.nlm.nih.gov/gene, accessed on 14 January 2025.

Gene	Forward Primer (5′-3′)	Forward Reverse (5′-3′)	Name/Function/Gene ID
*Atg10*	5′-TTC ACA GCA GAT AGG CGA TG-3′	5′-TGC AGG TCT CGT CAC TTC AG-3′	Autophagy related 10. Facilitates Atg12 conjugating enzyme activity. Gene ID: 66795
*Bag1*	5′-GAA ACA CCG TTG TCA GCA CT-3′	5′-GCT CCA CTG TGT CAC ACT C-3′	BCL2-associated athanogene 1. An oncogene involved in a pathway leading to apoptosis or programmed cell death. Gene ID: 12017
*Bag3*	5′-ATG GAC CTG AGC GAT CTC A-3′	5′-CAC GGG GAT GGG GAT GTA-3′	BCL2-associated athanogene 3. Enables chaperone binding activity. Gene ID: 29810
*Bassoon*	5′-CAG CCA GAG AAC AAC TTC TC-3′	5′-GTC CGA GGT CTT GCA TAT TG-3′	Structural component of presynaptic active zone. Gene ID: 12217
*Bche*	5′-ATG GGA GTG ATG CAT GGC TA-3′	5’-TCT CCA GAA CTG ACA TTG GG-3′	Butyrylcholinesterase. Facilitates acetylcholinesterase activity. Gene ID: 12038
*CatB*	5′-CTG CTT ACC ATA CAC CAT CC-3′	5′-ATC TCC TTC ACA CTG TTA GAC-3′	Cathepsin B. Lysosomal cysteine protease important for protein turnover. Gene ID: 13030
*CatD*	5′-CTG TAT CGG TTC CAT GTA AGT-3′	5′-CCA AGC ATT AGT TCT CCT CC-3′	Cathepsin D. Important for autophagosome assembly. Gene ID: 13033
*Gapdh*	5’ GTG TTT CCT CGT CCC GTA GA 3′	5′AAT CTC CAC TTT GCC ACT G 3′	Glyceraldehyde-3-phosphate dehydrogenase. Used as a housekeeping gene. Gene ID: 14433
*Herc1*	5′-TGA GTT GCT GCT TGG CTT AG-3′	5′-TTC TCT CCA CTG ACT CCG AT-3′	HECT and RLD domain containing E3 ubiquitin protein ligase family member 1. Involved in UPS activity. Gene ID: 235439
*Hspb8*	5′ ATA CGT GGA AGT TTC AGG CA 3′	5′ TCC TTT GAC CTA ACG CAA CC 3′	Heat shock protein 8. Important in cellular response to unfolded protein. Gene ID: 80888
*MAP-1lc3β*	5′ CGT CCT GGA CAA GAC CA 3′	5′ CCA TTC ACC AGG AGG AA 3′	Microtubule-associated protein 1 light chain 3 beta. Ubiquitin protein ligase binding activity; involved in autophagy. Gene ID: 67443
*Munc13*	5′-TGG AGT CAT GTC TCT GCT GT-3′	5′-TCC CAG ATG AGA CCC TTG TT-3′	Mammalian uncoordinated-13. Important for synaptic vesicle priming. Gene ID: 22249
*P62/Sqstm1*	5′-AGG GAA CAC AGC AAG CT-3′	5′-GCC AAA GTG TCC ATG TTT CA-3′	Sequestosome 1. Enables K63-linked polyubiquitin modification-dependent protein binding activity. Gene ID: 18412
*Psmc2*	5′ GGA CTG ATG CTT GCT TCA TTC 3′	5′ CAG CTG ATT GAT GAG CTC CAG 3′	Proteasome 26S subunit, ATPase 2. Important for proteasome activity. Gene ID: 19181
*Rab7*	5′-TGC TGA AGG TCA TCA TCC TG-3′	5′-AGA GAC TGG AAC CGT TCT TG-3′	Member RAS oncogene family. Involved in lipophagy. Gene ID: 19349
*S100β*	5′-TGC CCT CAT TAG TGT CTT CCA-3′	5′-GAG AGA GCT CGT TGT TGA TAA-3′	S100 protein, beta polypeptide, neural. Regulation of neuronal synaptic plasticity. Gene ID: 20203
*Syp*	5′-AGA CAT GGA CGT GGT GAA TC-3′	5′-GGA GGG TGC ATC AAA GTA CA-3′	Synaptophysin. Involved in synaptic transmission and synaptic vesicle endocytosis. Gene ID: 20977

## Data Availability

Data are contained within the article.
